# Fabry nephropathy as the clinical anchor for kidney-centered metabolic surveillance: linking genetic heterogeneity to early risk identification

**DOI:** 10.3389/fgene.2026.1905397

**Published:** 2026-08-04

**Authors:** Yanshu Xie, Jingzi Zhong

**Affiliations:** 1 The Clinical Laboratory Center, The First Affiliated Hospital of Guangxi Medical University, Nanning, Guangxi, China; 2 Department of Pediatrics, The First Affiliated Hospital of Guangxi Medical University, Nanning, Guangxi, China

**Keywords:** fabry disease, fabry nephropathy, genetic heterogeneity, GLA, kidney disease, lyso-Gb3, precision medicine, X-chromosome inactivation

## Abstract

Fabry disease is an X-linked lysosomal storage disorder caused by pathogenic variants in *GLA*, in which the kidney is a principal target organ and Fabry nephropathy is a major determinant of long-term outcome. Its genetic architecture is heterogeneous: *GLA* variant class, residual α-galactosidase A (α-Gal A) activity, lyso-Gb3 level (globotriaosylsphingosine, the deacylated metabolite of globotriaosylceramide [Gb3]), sex, age, X-linked mosaicism, renal vulnerability, and tissue susceptibility together shape renal and systemic phenotype and define the framework for precision surveillance. Fabry nephropathy serves as the clinical anchor for integrating endocrine and metabolic findings into precision surveillance. Rather than providing a broad Fabry disease review, we evaluate endocrine domains according to evidence strength and renal relevance. Thyroid dysfunction, reproductive health, and bone/vitamin D abnormalities currently have moderate support; pituitary findings, growth and puberty, and body composition remain limited; and glucose/lipid metabolism and inflammatory-metabolic profiling are exploratory. Endocrine and metabolic assessment should therefore be integrated into risk-adapted surveillance strategies alongside renal monitoring, particularly in genotype-positive children and females, in whom early disease trajectories and long-term organ involvement remain difficult to predict.

## 1 Introduction

Fabry disease is an X-linked inherited metabolic disorder caused by pathogenic variants in *GLA*, which encodes α-galactosidase A (α-Gal A). Deficient α-Gal A activity leads to lysosomal accumulation of globotriaosylceramide (Gb3) and its deacylated metabolite globotriaosylsphingosine (lyso-Gb3). Although glycosphingolipid storage remains central to disease biology, clinical expression varies substantially according to variant class, residual enzyme activity, lyso-Gb3 level, sex, age, and tissue susceptibility (Lukas et al., 2013; Arends et al., 2017; Lenders and Brand, 2021). This review therefore uses Fabry disease as a model of genetically driven but biologically modified endocrine and metabolic risk.

Among the major target organs affected by Fabry disease, the kidney remains one of the most clinically important determinants of long-term morbidity and treatment decisions. However, endocrine and metabolic abnormalities may emerge before advanced Fabry nephropathy becomes evident and could provide complementary information regarding systemic vulnerability, nutritional status, inflammatory burden, mineral metabolism, and potentially modifiable risk factors. The strongest endocrine signals currently concern thyroid dysfunction, fertility/reproductive health, and bone or vitamin D status, whereas pituitary findings, growth and puberty, body composition, and glucose/lipid metabolism require more cautious interpretation (Bothou et al., 2022; Varaldo et al., 2025). This uneven evidence base is especially relevant in females and genotype-positive children, where renal risk, X-linked mosaicism, residual enzyme activity, and longitudinal penetrance must be interpreted together. Children and females are emphasized because presymptomatic detection, age-dependent penetrance, and X-linked mosaicism make risk interpretation particularly challenging, although the framework may also inform adult male surveillance.

Previous reviews have summarized endocrine abnormalities in Fabry disease, including thyroid dysfunction, reproductive health, pituitary findings, and skeletal involvement. The objective of the present review is different. Rather than cataloging endocrine manifestations, we integrate genetic heterogeneity, female X-linked mosaicism, Fabry nephropathy, and pediatric/presymptomatic surveillance into a kidney-centered framework. We further introduce qualitative evidence grading and renal relevance assessment to distinguish endocrine domains that may contribute to longitudinal risk stratification from those that currently remain exploratory. In this regard, the review aims to move from description of endocrine findings toward a clinically oriented surveillance model.

**FIGURE 1:**
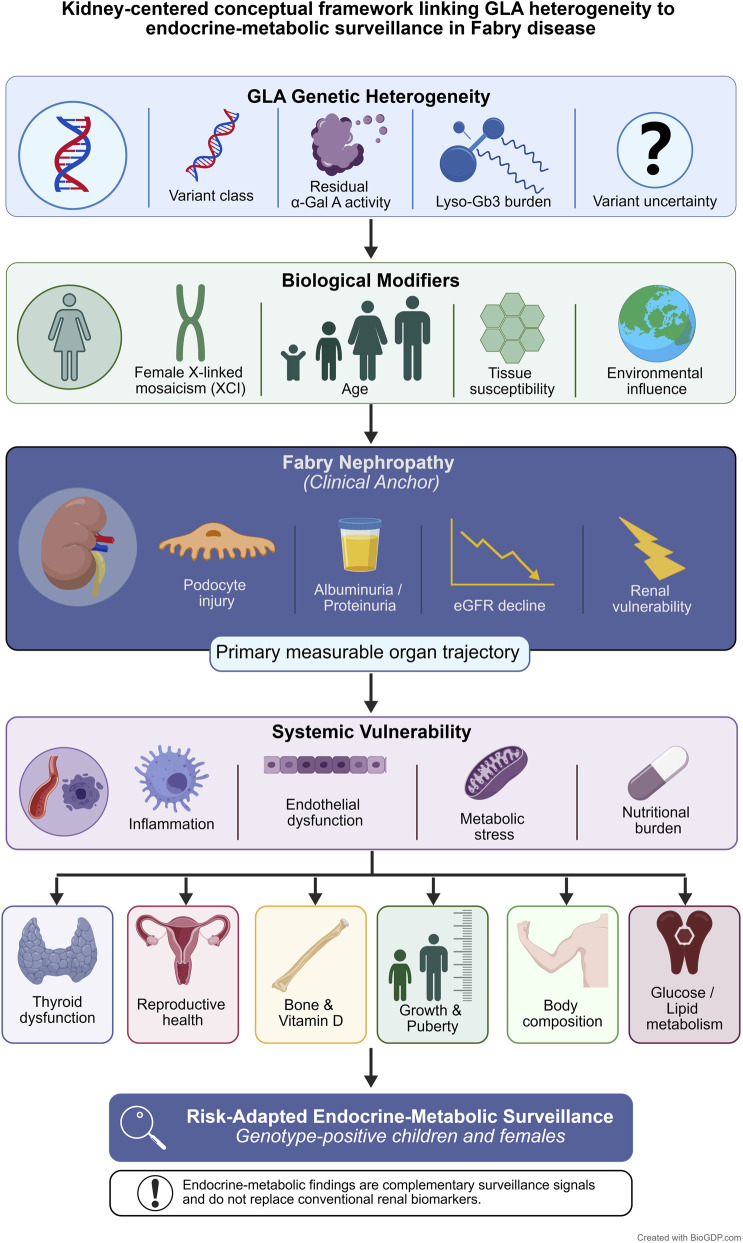
Kidney-centered conceptual framework linking GLA heterogeneity to metabolic surveillance in Fabry disease. Genetic and biochemical modifiers shape renal vulnerability, while selected endocrine and metabolic domains are interpreted according to evidence strength and renal relevance.

This review proposes a kidney-centered framework in which Fabry nephropathy serves as the clinical anchor linking genetic heterogeneity to metabolic surveillance. The conceptual framework proposed in this review is summarized in Figure 1(Jiang et al., 2025).

## 2 Literature search and evidence classification

We searched PubMed/MEDLINE and Google Scholar for English-language articles published up to May 2026 using combinations of the following terms: “Fabry disease”, “*GLA*”, “Fabry nephropathy”, “kidney”, “albuminuria”, “eGFR”, “lyso-Gb3”, “female”, “X-chromosome inactivation”, “children”, “newborn screening”, “thyroid”, “fertility”, “gonadal”, “bone mineral density”, “vitamin D”, “growth”, “puberty”, “metabolism”, and “inflammation”. Priority was given to consensus statements, cohort studies, registry analyses, mechanistic studies, and recent reviews directly relevant to renal outcomes or endocrine and metabolic surveillance. Case reports and small series were included when evidence was sparse. This was a narrative review rather than a systematic review; therefore, no formal risk-of-bias assessment or meta-analysis was performed.

For the purposes of this review, evidence strength was classified qualitatively. “Moderate” evidence indicates findings supported by multiple cohort studies, prospective studies, registry analyses, or consistent observational data. “Limited” evidence indicates findings supported mainly by small cohorts, case series, cross-sectional studies, or inconsistent observations. “Exploratory” evidence indicates preliminary clinical associations, mechanistic hypotheses, biomarker studies, or findings lacking sufficient validation for routine surveillance. Mechanistic evidence was classified separately as “direct”, “indirect”, or “speculative”. “Direct” evidence refers to mechanisms demonstrated in Fabry disease tissues, disease-specific cellular or animal models, or well-established renal disease pathways. “Indirect” evidence refers to biologically plausible mechanisms supported by related experimental or clinical observations but lacking direct endocrine-organ validation. “Speculative” evidence refers to hypotheses with limited supporting data that require further investigation. These categories were intended to provide a pragmatic clinical framework rather than a formal GRADE-based evidence assessment. They were not intended to function as GRADE-based certainty ratings, but to help clinicians distinguish domains with relatively consistent clinical support from those that remain hypothesis-generating.

## 3 Genetic basis and genotype–phenotype heterogeneity

Fabry disease is caused by pathogenic *GLA* variants, but *GLA* genotype alone is not sufficient to predict phenotype, renal trajectory, or surveillance needs. Genotype provides the molecular starting point, whereas residual α-Gal A activity, lyso-Gb3 level, sex-specific modifiers, family segregation, renal findings, organ vulnerability, and longitudinal evolution together determine clinical expression. For this focused review, the relevant genetic issue is therefore not to catalog every Fabry phenotype, but to identify which molecular and biochemical features should inform a kidney-centered surveillance framework (Lukas et al., 2013; Arends et al., 2017; Lenders and Brand, 2021).

The *GLA* variant spectrum includes missense, nonsense, frameshift, splice-site, intronic, insertion/deletion, and rearrangement variants (Mehta and Hughes, 2002; Arends et al., 2017). Missense variants may alter protein folding, stability, trafficking, or catalytic function, whereas nonsense, frameshift, and severe splice defects more often produce marked loss of enzyme function. RNA-processing defects and noncanonical splicing can further modify genotype–phenotype interpretation when coding or intronic variants alter transcript structure (Alfen et al., 2022).

Residual α-Gal A activity and lyso-Gb3 level form the principal biochemical bridge between genotype and phenotype. Residual α-Gal A activity helps estimate the functional impact of a *GLA* variant, but it is not deterministic, especially in females, where X-linked mosaicism may produce normal or borderline enzyme activity despite clinically meaningful disease (Juchniewicz et al., 2018; Faro et al., 2023). Lyso-Gb3 level provides complementary biochemical information about substrate-related disease burden and may help distinguish classic from later-onset phenotypes or support interpretation of low-penetrance and uncertain variants. However, lyso-Gb3 level should be interpreted with genotype, residual α-Gal A activity, sex, family segregation, renal findings, and longitudinal phenotype rather than used as a stand-alone marker (Niemann et al., 2014; Nowak et al., 2022; Ramaswami et al., 2025).

Classic, late-onset, and organ-predominant Fabry disease should be viewed as a continuum rather than rigid categories. Classic disease is usually associated with very low residual α-Gal A activity and early multisystem involvement, whereas late-onset forms retain more residual α-Gal A activity and may present with cardiac-, renal-, or other organ-predominant phenotypes (Mehta and Hughes, 2002; Arends et al., 2017; Spada et al., 2017). In this continuum, renal trajectory is particularly useful for surveillance because albuminuria, proteinuria, eGFR change, and blood pressure provide measurable longitudinal signals that can help determine when molecular risk becomes clinically actionable.

Sequencing and screening have increased detection of variants of uncertain significance (VUS), pseudodeficiency alleles, debated variants, and late-onset-associated alleles (Mehta and Hughes, 2002; Germain et al., 2022). These findings should not be equated automatically with clinical Fabry disease. Interpretation should integrate residual α-Gal A activity, lyso-Gb3 level, population frequency, family segregation, organ findings, renal trajectory, and functional evidence when available, so that *GLA*-positive status is not overinterpreted as clinically manifest disease (Lenders et al., 2019; Germain et al., 2022).

Migalastat amenability is a treatment-relevant genetic feature and should therefore be considered after basic variant classification and biochemical interpretation. Amenability should be based on validated data rather than inferred from missense status alone, because not all missense variants encode proteins that respond to pharmacological chaperone therapy (Benjamin et al., 2017; Lenders and Brand, 2021). Endocrine improvement is not currently a validated response endpoint, so migalastat status should inform treatment eligibility rather than define endocrine or metabolic surveillance intensity.

Taken together, *GLA* genotype is the starting point for Fabry disease risk stratification, but it must be integrated with biochemical, renal, family, and longitudinal data. Residual α-Gal A activity and lyso-Gb3 level help translate genotype into biological risk, whereas renal findings and follow-up trajectories determine whether risk is becoming clinically actionable. This integrated approach supports precision diagnosis and avoids both under-recognition of Fabry disease and overdiagnosis based on genotype alone.

## 4 Female heterogeneity and X-chromosome inactivation

Female Fabry disease illustrates why *GLA* genotype alone cannot predict clinical expression. Heterozygous females should not be described as passive “carriers”; they have an X-linked mosaic disorder in which the proportion and distribution of cells expressing the pathogenic *GLA* allele can vary across tissues (Echevarria et al., 2016; Izhar et al., 2023). Clinical severity ranges from asymptomatic biochemical abnormality to meaningful neuropathic pain, autonomic/gastrointestinal symptoms, cerebrovascular manifestations, cardiac, renal, and quality-of-life involvement. For this review, the key implication is that female endocrine and metabolic findings should be interpreted within a mosaic disease model rather than dismissed as unrelated comorbidity.

X-chromosome inactivation (XCI) is an important modifier but not a stand-alone prognostic test. Skewing toward inactivation of the normal X chromosome may increase the burden of cells expressing mutant *GLA*, and several studies link skewed XCI with more severe expression (Echevarria et al., 2016; Juchniewicz et al., 2018; Wagenhäuser et al., 2022). However, blood XCI may not mirror myocardium, kidney, endothelium, nervous tissue, or endocrine organs; methods are not harmonized; and organ injury reflects cumulative substrate exposure, vascular injury, inflammation, age, and other modifiers. XCI should therefore be used as one component of risk interpretation, not as a single clinical decision rule.

Female-specific endocrine involvement remains insufficiently defined. Thyroid function, gonadal axis, fertility, vitamin D status, bone health, body composition, and metabolic markers deserve systematic evaluation, but available studies are small and rarely stratified by sex, *GLA* variant, lyso-Gb3 level, or XCI pattern (Faggiano et al., 2006; Maione et al., 2015). Endocrine assessment in women should currently be framed as risk-adapted surveillance rather than as a validated female-specific Fabry phenotype.

In females, α-galactosidase A activity and lyso-Gb3 level may be normal, borderline, or variably abnormal because of X-linked mosaicism (Mehta and Hughes, 2002; Duro et al., 2024). Diagnosis and follow-up should integrate *GLA* sequencing, biochemical testing, family history, symptoms, cardiac and renal surveillance, and metabolic surveillance when clinically relevant.

Overall, female Fabry disease is genetically determined, mosaic, and dynamically modified by XCI, residual enzyme activity, lyso-Gb3 level, age, epigenetic state, and organ-specific susceptibility. This framework supports longitudinal, sex-informed surveillance and reinforces the need to integrate renal and endocrine and metabolic assessment in female Fabry disease.

## 5 Fabry nephropathy as the clinical anchor for metabolic surveillance

Fabry nephropathy provides the clinical anchor for a kidney-centered metabolic surveillance model. The kidney is one of the major target organs in Fabry disease and remains central to long-term morbidity, treatment timing, and therapeutic response assessment. In practice, renal involvement often determines whether genotype-positive patients move from molecular risk to clinically actionable disease. Podocyte injury, albuminuria or proteinuria, progressive decline in estimated glomerular filtration rate (eGFR), and hypertension form the key renal trajectory that clinicians follow over time (Sanchez-Niño et al., 2011; Germain et al., 2019; Nowak et al., 2022). These measures are not merely organ-specific outcomes; they also reflect cumulative substrate burden, microvascular injury, inflammation, and tissue remodeling.

Although Fabry disease is also a major cause of cardiac involvement, nephropathy is particularly suitable for this role. Albuminuria and proteinuria can emerge early, eGFR trajectories are quantifiable across longitudinal follow-up, and treatment decisions frequently depend on renal stage, renal reserve, and evidence of progressive kidney injury. In genotype-positive children, renal surveillance may identify early transition from molecular risk to organ involvement without waiting for advanced systemic disease. In females, where enzyme activity and lyso-Gb3 level may be less reliable because of X-linked mosaicism, albuminuria and eGFR trajectories are established markers of Fabry nephropathy and disease progression. Blood pressure measurements provide additional clinically actionable information during longitudinal surveillance. This does not diminish the importance of cardiac disease; rather, it explains why a kidney-centered framework is useful for early, measurable, and modifiable risk identification. Recent real-world studies further support the importance of longitudinal renal monitoring in Fabry disease. A 20-year single-center observational cohort described long-term clinical outcomes in adults with Fabry disease, while data from a national Fabry center identified epidemiological features and early predictors of Fabry nephropathy that are directly relevant to renal risk stratification and precision follow-up (Mannan et al., 2025; McCarron et al., 2025).

Within this model, endocrine and metabolic abnormalities are interpreted after the renal trajectory has been defined. Thyroid dysfunction, vitamin D deficiency, mineral imbalance, altered body composition, reproductive concerns, fatigue, dyslipidemia, and inflammatory-metabolic changes should be interpreted as complementary signals. They may clarify systemic burden, nutritional resilience, vascular risk, and modifiable contributors to kidney outcomes. This is most useful when renal trajectories are early, subtle, or confounded by age, sex, treatment exposure, or comorbid metabolic risk.

This kidney-centered framing is especially relevant for children and females. Genotype-positive children may be identified before albuminuria or eGFR decline becomes evident. They therefore need structured follow-up without overmedicalization of molecular risk. Female patients may have normal or borderline α-galactosidase A activity and variable lyso-Gb3 level because of X-linked mosaicism. Renal or systemic involvement may still develop. In both groups, risk interpretation should follow a stepwise sequence: *GLA* heterogeneity, biological modifiers, nephropathy, systemic vulnerability, endocrine and metabolic signals, and longitudinal follow-up.

## 6 Mechanistic links between fabry nephropathy and endocrine and metabolic vulnerability

The mechanistic question is not simply how Fabry disease affects endocrine tissues, but how established pathways leading to Fabry nephropathy may also create broader endocrine and metabolic vulnerability. Because renal injury represents one of the best-characterized manifestations of Fabry disease, kidney-related mechanisms provide a useful biological framework for interpreting endocrine and metabolic findings. Direct endocrine-organ evidence remains limited, so mechanisms should be graded as direct, indirect, or speculative. Three overlapping axes are most relevant. Mechanistic evidence was classified as direct, indirect, or speculative according to the strength of Fabry-specific experimental and clinical support, as defined above.

### 6.1 Lysosomal-storage axis

Deficient α-galactosidase A activity causes lysosomal accumulation of globotriaosylceramide (Gb3)-related substrates and contributes directly to renal cellular injury, particularly in podocytes and other kidney cell populations. The same lysosomal and endoplasmic reticulum (ER) stress pathways may also increase endocrine and metabolic vulnerability. Disrupted cellular trafficking, autophagy, nutrient sensing, organelle quality control, ER stress, and unfolded protein response pathways link variant biology to cell injury beyond storage alone (Kim et al., 2021; Li et al., 2024; Lenders et al., 2025). Evidence level: direct for lysosomal storage, ER stress, and autophagy disruption in Fabry models; indirect or speculative for endocrine-organ consequences.

### 6.2 Vascular-inflammatory axis

The vascular-inflammatory axis is most clearly illustrated in Fabry nephropathy, where lyso-Gb3 accumulation contributes to podocyte dysfunction, albuminuria or proteinuria, progressive glomerular injury, and subsequent decline in renal function (Sanchez-Niño et al., 2011; Hwang et al., 2023; Ramaswami et al., 2025). Similar pathways may influence endocrine and metabolic tissues through vascular injury, complement activation, cytokine signaling, oxidative stress, fibrosis, and reduced microvascular reserve (Choi et al., 2023; Faro et al., 2024; Kurdi et al., 2024). Thus, renal tissue provides a biologically relevant bridge between systemic disease burden and endocrine and metabolic vulnerability. Evidence level: direct for systemic vascular/inflammatory and renal injury; indirect for endocrine consequences; speculative for endocrine fibrosis as a distinct Fabry process.

### 6.3 Metabolic stress axis

Mitochondrial dysfunction and oxidative stress have been implicated in renal cellular injury and progressive Fabry nephropathy, and may also contribute to broader endocrine and metabolic abnormalities. These downstream features of lysosomal disease have been linked to altered energetics, calcium handling, and reactive oxygen species in Fabry models (Das and Naim, 2009; Elsaid et al., 2023; Faro et al., 2024). Steroidogenesis, insulin secretion, thyroid hormone synthesis, and bone remodeling are metabolically demanding processes. Evidence level: indirect; clinical endocrine abnormalities have been reported, but mechanistic mitochondrial links remain speculative.

## 7 Endocrine and metabolic manifestations

Beyond their clinical relevance as individual manifestations, endocrine and metabolic abnormalities may provide complementary information regarding systemic burden and renal vulnerability in Fabry disease. The key question is not simply whether these abnormalities occur, but whether they contribute to longitudinal risk assessment alongside established renal biomarkers. Because endocrine data in Fabry disease are heterogeneous, the following subsections distinguish relatively documented associations, such as thyroid dysfunction, reproductive health, bone density, and vitamin D status, from exploratory areas such as growth, puberty, glucose metabolism, and inflammatory-metabolic profiling. This distinction is important because the endocrine phenotype is unlikely to result from a single mechanism. Instead, it probably reflects interactions among glycosphingolipid storage, lyso-Gb3 signaling, microvascular dysfunction, chronic inflammation, renal impairment, reduced mobility, nutritional factors, sex-specific biology, and treatment exposure (Faggiano et al., 2006; Bothou et al., 2022). Endocrine and metabolic findings should therefore be interpreted as potentially informative components of Fabry surveillance rather than as universally present disease-defining manifestations.

Table 1 summarizes the qualitative evidence classification, representative database identifiers, and renal relevance of endocrine and metabolic in Fabry disease.

**TABLE 1:** Qualitative evidence classification and renal relevance of endocrine and metabolic domains in Fabry disease.

Domain	Represent active database identifier(s)	Evidence	Evidence source	Renal relevance	Suggested monitoring
Renal vulnerability	KEGG Disease: H00125 Fabry disease; GO:0003014 renal system process; GO:0003094 glomerular filtration	Core clinical anchor	Consensus; mechanistic and cohort studies (Mehta and Hughes, 2002; Nowak et al., 2022; Sanchez-Niño et al., 2011; Germain et al., 2019; McCarron et al., 2025; Mannan et al., 2025)	Albuminuria and eGFR trajectory define nephrology risk and contextualize endocrine and metabolic findings	Albuminuria/proteinuria, eGFR, blood pressure, lyso-Gb3 level, and renal history alongside endocrine surveillance
Thyroid dysfunction	GO:0042403 thyroid hormone metabolic process	Moderate	Cohort studies (Bothou et al., 2022; Hauser et al., 2005; Faggiano et al., 2011)	Hypothyroidism may aggravate dyslipidemia, fatigue, weight change, and chronic kidney disease (CKD)-associated metabolic burden	Baseline and periodic TSH/free T4, especially with fatigue, weight change, dyslipidemia, growth concerns, or declining kidney function
Fertility/reproductive health	GO:0022414 reproductive process	Moderate	Cohort studies; case series (Faggiano et al., 2006; Papaxanthos-Roche et al., 2019; Laney et al., 2017; Haninger-Vacariu et al., 2024)	Pregnancy planning and reproductive decisions require renal risk assessment, especially with albuminuria, CKD, or hypertension	Reproductive counseling; gonadal hormones or semen analysis when indicated; preconception renal and cardiac assessment
Bone/vitamin D/mineral metabolism	GO:0030282 bone mineralization	Moderate	Cohort studies; case series (Varaldo et al., 2025; Bruell et al., 2022)	Mineral metabolism interacts closely with Fabry nephropathy, CKD progression, and chronic kidney disease-mineral and bone disorder (CKD-MBD) pathways	25-hydroxyvitamin D, calcium-phosphate-parathyroid hormone (PTH) profile, renal function, and dual-energy X-ray absorptiometry (DXA)/trabecular bone score (TBS) in selected risk groups
Pituitary morphology/function	GO:0042445 hormone metabolic process; GO:0046883 regulation of hormone secretion	Limited	Small cohort studies (Maione et al., 2015)	Potential effects on fluid balance, fatigue, growth, or reproductive axes remain poorly linked to renal outcomes	No universal screening; test pituitary axes when symptoms or imaging findings suggest dysfunction
Growth/puberty/body composition	GO:0040007 growth	Limited	Pediatric cohorts; case series (Lu et al., 2023)	Sarcopenia, poor nutrition, and delayed growth may influence renal resilience and CKD outcomes	Pediatric growth velocity, puberty, BMI, nutrition, and muscle/function assessment when feasible
Glucose/lipid metabolism	GO:0006006 glucose metabolic process; GO:0006629 lipid metabolic process; KEGG: hsa00600 sphingolipid metabolism	Exploratory	Observational data (Biddeci et al., 2025)	Dyslipidemia, insulin resistance, and cardiometabolic risk may compound CKD and vascular risk	Risk-adapted glucose/HbA1c, lipid profile, blood pressure, body composition, and lifestyle assessment
Inflammatory-metabolic profiling	GO:0006954 inflammatory response	Exploratory	Research studies (Kurdi et al., 2024; Biddeci et al., 2025)	Inflammation and endothelial stress may link Fabry nephropathy with systemic metabolic vulnerability	Research or registry setting; define thresholds before routine clinical use

Database identifiers are representative ontology or pathway terms selected to map each clinical domain to broadly relevant biological processes or disease/pathway categories. They are not intended to imply pathway enrichment analysis or domain-specific molecular validation.

Evidence categories were defined qualitatively for this narrative review. Representative references supporting each classification are shown as anchor studies rather than an exhaustive evidence inventory.

### 7.1 Growth and pubertal development

Growth and pubertal development represent clinically important but still exploratory areas in pediatric Fabry disease. Children may experience neuropathic pain, hypohidrosis, heat or exercise intolerance, gastrointestinal symptoms, fatigue, and reduced physical activity before advanced renal or cardiac injury becomes evident (Germain et al., 2019). These symptoms can indirectly affect nutrition, sleep, physical activity, school participation, and psychosocial wellbeing. However, direct evidence for impaired linear growth or abnormal pubertal timing as consistent Fabry manifestations remains limited. Growth delay, delayed puberty, or abnormal pubertal progression should therefore prompt evaluation for common pediatric endocrine, nutritional, gastrointestinal, inflammatory, or renal causes rather than be attributed automatically to Fabry disease.

Nevertheless, pediatric metabolic surveillance is justified. Low skeletal muscle mass has been reported as a possible early sign in children with Fabry disease, supporting the concept that systemic body composition and nutritional status may be affected before advanced organ disease develops (Lu et al., 2023). Growth velocity, body mass index, pubertal staging, nutritional intake, muscle mass, exercise tolerance, pain burden, and gastrointestinal symptoms should be monitored, especially in children with classic genotypes, elevated lyso-Gb3 level, chronic gastrointestinal complaints, renal involvement, or pain-related inactivity.

### 7.2 Thyroid dysfunction

Thyroid dysfunction is one of the relatively better documented endocrine findings in Fabry disease. Earlier studies reported a high prevalence of subclinical hypothyroidism in patients with Anderson–Fabry disease, raising the possibility that thyroid involvement may be part of the broader multisystem phenotype (Hauser et al., 2005). Subsequent work evaluating thyroid function before and after enzyme replacement therapy suggested that thyroid abnormalities may occur in Fabry disease, although the effect of disease-specific therapy on thyroid status remains uncertain (Faggiano et al., 2011). Prospective endocrine evaluation has also supported the presence of thyroid-related abnormalities in Fabry cohorts (Bothou et al., 2022).

The mechanisms underlying thyroid dysfunction are probably multifactorial. Possible contributors include lysosomal dysfunction in thyroid follicular cells, microvascular injury, inflammation, renal disease, altered hormone metabolism, iodine status, and coincidental autoimmune thyroid disease. Pediatric data are sparse; therefore, thyroid surveillance should be risk-adapted rather than assumed mandatory at high frequency for every patient. In practice, thyroid-stimulating hormone and free thyroxine may be included in baseline and periodic assessment, particularly in patients with fatigue, growth concerns, menstrual irregularity, weight change, dyslipidemia, or unexplained decline in quality of life. From a nephrology perspective, these features matter because fatigue, weight change, and dyslipidemia may complicate interpretation of CKD risk, treatment tolerance, and quality-of-life changes in patients with Fabry nephropathy.

### 7.3 Gonadal axis and fertility

Reproductive health is another clinically relevant domain with moderate but still incomplete evidence. Fabry disease may influence fertility and gonadal function through vascular dysfunction, chronic inflammation, renal impairment, autonomic dysfunction, pain, fatigue, medication exposure, and possible direct effects on reproductive tissues (Sansone et al., 2024). Early endocrine studies reported hormonal and fertility-related abnormalities in patients with Anderson–Fabry disease (Faggiano et al., 2006). The FERTIFABRY multicenter observational study provided more specific evidence by assessing semen and male genital tract characteristics, suggesting that semen parameters may be altered in some male patients (Papaxanthos-Roche et al., 2019). Studies on reproductive fitness further indicate that Fabry disease may affect family planning, reproductive decision-making, and psychosocial outcomes (Laney et al., 2017).

Female reproductive health requires separate consideration because heterozygous females may develop clinically meaningful disease. Available pregnancy studies and case reports indicate that successful pregnancies are possible, including in selected women receiving enzyme replacement therapy, but pregnancy should be managed individually according to cardiac, renal, neurological, and obstetric risk (Haninger-Vacariu et al., 2024). Evidence on ovarian reserve, menstrual function, and female gonadal axis involvement remains limited. Therefore, reproductive care should include genetic counseling, discussion of X-linked inheritance, preconception cardiac and renal assessment, review of treatment exposure, and multidisciplinary coordination among metabolic specialists, obstetricians, nephrologists, cardiologists, and reproductive endocrinologists when needed. Pregnancy planning should explicitly incorporate albuminuria or proteinuria, eGFR, blood pressure, cardiac status, and medication exposure, because renal reserve strongly influences maternal and fetal risk.

### 7.4 Bone, vitamin D, and mineral metabolism

Bone and mineral metabolism represent one of the more documented endocrine and metabolic domains in Fabry disease. Osteopenia, osteoporosis, reduced bone mineral density, altered trabecular bone score, vitamin D deficiency, and mineral metabolism disturbances have been reported, but their mechanisms are likely multifactorial (Bruell et al., 2022; Varaldo et al., 2025). Potential contributors include reduced physical activity due to pain or fatigue, gastrointestinal burden, hypogonadism, renal impairment, chronic inflammation, vitamin D deficiency, and altered calcium-phosphate-PTH regulation. Bone health should therefore be considered part of long-term multidisciplinary care rather than an incidental comorbidity.

Because kidney function is central to vitamin D activation and mineral homeostasis, skeletal abnormalities in Fabry disease should also be interpreted within the context of Fabry nephropathy. Interactions among renal dysfunction, vitamin D deficiency, altered calcium-phosphate balance, and chronic inflammation may contribute substantially to long-term skeletal risk.

Clinically, assessment of 25-hydroxyvitamin D, calcium, phosphate, PTH, alkaline phosphatase, renal function, and bone mineral density may identify modifiable contributors to skeletal risk. In children and adolescents, vitamin D and mineral status deserve particular attention because peak bone mass accrual occurs during puberty and early adulthood. These parameters should be interpreted together with pubertal stage, nutrition, physical activity, renal status, pain, and gastrointestinal symptoms.

### 7.5 Glucose/lipid metabolism and systemic inflammation

Glucose and lipid metabolism remain exploratory areas in Fabry disease. Although Fabry disease is a glycosphingolipid disorder, current evidence does not support diabetes mellitus as a typical Fabry manifestation. Lipid and inflammatory changes may reflect broader sphingolipid disruption, endothelial dysfunction, reduced mobility, renal impairment, and chronic inflammatory burden rather than a primary endocrine phenotype. Whether such signatures predict endocrine or metabolic outcomes remains uncertain. Although these findings are not typical Fabry manifestations, they may compound vascular-renal risk and should be considered when interpreting CKD progression or cardiovascular risk in Fabry nephropathy.

Systemic inflammation may further shape metabolic risk. Chronic inflammatory and immune pathways, endothelial dysfunction, oxidative stress, reduced mobility, dietary limitations, and renal dysfunction may influence insulin sensitivity, lipid profiles, muscle metabolism, bone remodeling, and cardiovascular risk (Biddeci et al., 2025). Therefore, metabolic assessment should include glucose or HbA1c when clinically indicated, lipid profile, body composition, renal function, blood pressure, inflammatory context, physical activity, and diet. These assessments are useful for precision surveillance even when causality is not fully established.

### 7.6 Pediatric endocrine surveillance

No universally accepted Fabry-specific pediatric endocrine surveillance guideline currently exists, but a proactive and risk-adapted approach is reasonable. Pediatric consensus recommendations emphasize early recognition, structured follow-up, and individualized management in children with Fabry disease (Germain et al., 2019). For genotype-confirmed pediatric patients, endocrine and metabolic surveillance may include serial height, weight, body mass index, growth velocity, pubertal staging, nutritional assessment, muscle mass or functional capacity when available, thyroid function, vitamin D status, calcium-phosphate-PTH profile, renal function, albuminuria or proteinuria, lipid profile, glucose or HbA1c when indicated, and assessment of pain, fatigue, gastrointestinal symptoms, and physical activity.

Surveillance intensity should be higher in children with classic disease, elevated lyso-Gb3 level, early symptoms, chronic gastrointestinal burden, reduced mobility, renal involvement, inflammatory features, or family history of severe disease. In adolescents, reproductive development, menstrual history, fertility counseling, and psychosocial impact should be addressed sensitively. Overall, endocrine and metabolic manifestations should be incorporated into a precision Fabry care model. Rather than treating thyroid dysfunction, fertility issues, bone disease, vitamin D deficiency, or metabolic abnormalities as unrelated comorbidities, clinicians should evaluate whether they reflect Fabry-related lysosomal, vascular, inflammatory, renal, nutritional, or treatment-associated mechanisms. This kidney-centered sequence is particularly relevant for pediatric and female patients, where early symptoms may be subtle and risk interpretation depends on genotype, renal trajectory, and systemic context.

## 8 Pediatric and presymptomatic screening

Newborn screening, next-generation sequencing, and genomic newborn screening can identify *GLA* variants before symptoms appear (Spada et al., 2006; Gragnaniello et al., 2023; Wang et al., 2025). This creates an opportunity for early surveillance, but also a risk of overdiagnosis because genetic detection does not automatically equal clinical Fabry disease. The purpose of pediatric screening is not immediate treatment for all genotype-positive children, but longitudinal risk stratification.

Newborn and genomic screening often detect *GLA* variants more frequently than expected from traditional clinical incidence estimates (Colon et al., 2017; Wang et al., 2025). Many findings represent late-onset, low-penetrance, debated, pseudodeficiency-associated, or uncertain variants rather than classic childhood-onset Fabry disease. Screening should therefore be framed as detection of molecular risk requiring interpretation, counseling, and follow-up.

For pediatric practice, the central distinction is between classic childhood-risk genotypes and late-onset or uncertain variants. This distinction helps avoid both delayed recognition of truly affected children and premature disease labeling in children whose risk may remain low or unclear for decades.

Overdiagnosis is the major challenge. Counting VUS, debated alleles, or late-onset variants as equivalent to pathogenic childhood Fabry disease can inflate prevalence estimates and expose families to anxiety, repeated testing, premature treatment discussions, and long-term uncertainty (van der Tol et al., 2014; Palaiodimou et al., 2022; Monda et al., 2023).

Children identified through screening should be stratified using genotype, α-galactosidase A activity, lyso-Gb3 level, family segregation, sex, early symptoms, and longitudinal organ assessment. No single result should determine disease status in isolation; variant class, enzyme activity, substrate burden, family history, and evolving clinical signs should be interpreted together.

Children with confirmed pathogenic or likely pathogenic *GLA* variants should enter structured follow-up after confirmation, but intensity should be risk-adapted (Mehta and Hughes, 2002; Hopkin et al., 2016; Germain et al., 2019). Classic male disease, very low enzyme activity, elevated lyso-Gb3 level, neuropathic pain, gastrointestinal symptoms, angiokeratomas, cornea verticillata, albuminuria, or severe family history justify closer surveillance and early treatment discussion; late-onset or uncertain variants require systematic but less intensive monitoring.

Baseline assessment should include genotype review, family history, symptoms, physical examination, pain/autonomic and gastrointestinal review, urinalysis, albuminuria/proteinuria, eGFR, blood pressure, ophthalmologic and hearing evaluation, and cardiac testing when clinically indicated (Mehta and Hughes, 2002; Hopkin et al., 2016; Germain et al., 2019). Follow-up intervals should track phenotype, biomarker trajectory, sex, family history, and variant classification.

Endocrine and metabolic indicators are not Fabry-specific diagnostic markers, but they may help identify systemic burden or modifiable morbidity in pediatric follow-up. Growth velocity, pubertal staging, nutrition, body composition or muscle function, thyroid function, vitamin D, calcium-phosphate-PTH profile, and selected bone-health measures are most relevant in children with classic genotypes, elevated lyso-Gb3 level, gastrointestinal burden, pain-related inactivity, or reduced muscle mass (Lu et al., 2023).

Presymptomatic diagnosis creates ethical and psychosocial challenges. Families need clear counseling about variant classification, age-dependent penetrance, the difference between genetic risk and clinical disease, and the rationale for longitudinal surveillance (Macklin et al., 2018).

In the genomic screening era, pediatric Fabry care must balance early recognition against overmedicalization. Metabolic surveillance can add a low-burden surveillance layer, but it should support longitudinal risk stratification rather than convert every genotype-positive child into an immediately treated patient.

## 9 Precision surveillance and future directions

**FIGURE 2:**
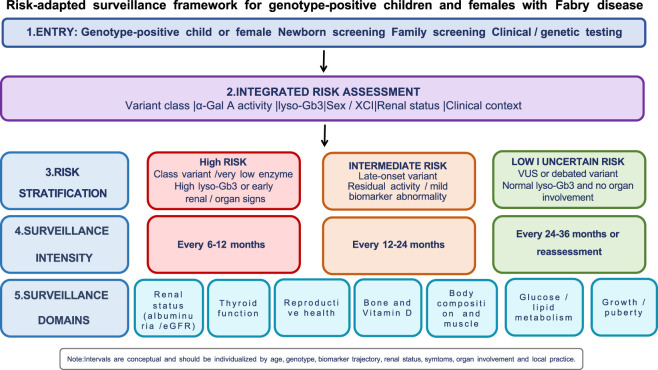
Risk-adapted surveillance framework for genotype-positive children and females with Fabry disease. Monitoring intensity is guided by variant interpretation, α-Gal A activity, lyso-Gb3 level, renal trajectory, symptoms, sex-specific factors, and treatment exposure.

The final task is to translate *GLA* heterogeneity, biological modifiers, and renal trajectory into a low-burden monitoring strategy. A practical risk-adapted surveillance framework for genotype-positive children and females is presented in Figure 2. The framework integrates genetic, biochemical, renal, and clinical features to support individualized longitudinal monitoring while avoiding overmedicalization of molecular risk.

### 9.1 Pediatric surveillance

In genotype-positive children, surveillance should be driven by molecular risk and renal trajectory rather than genotype alone (Germain et al., 2019; Wang et al., 2025). Variant class, residual α-Gal A activity, lyso-Gb3 level, family history, symptoms, albuminuria or proteinuria, eGFR, and blood pressure should define surveillance intensity. Endocrine and metabolic assessment should remain targeted, focusing on growth velocity, pubertal development, nutrition, body composition or muscle function, thyroid function, vitamin D status, and calcium-phosphate-PTH profile when clinically indicated.

### 9.2 Female surveillance

Female Fabry disease requires surveillance that treats heterozygosity as X-linked mosaic disease rather than carrier status (Wagenhäuser et al., 2022; Izhar et al., 2023; Brand et al., 2025). X-chromosome inactivation may inform risk interpretation but should not be used as a stand-alone decision tool. In females, renal trajectory, lyso-Gb3 level, symptoms, reproductive history, and selected endocrine or metabolic findings should be interpreted together to distinguish Fabry-related involvement from age-related, sex-specific, or coincidental comorbidity.

### 9.3 Registry endpoints

Future registries and cohort studies should prospectively capture renal-genetic context and endocrine or metabolic endpoints within the same data structure. Core data elements should include *GLA* variant class, α-galactosidase A activity, lyso-Gb3 level, albuminuria or proteinuria, eGFR, blood pressure, treatment exposure, and patient-reported outcomes. Selected endocrine and metabolic variables should include growth and puberty in children, thyroid function, reproductive and pregnancy outcomes, vitamin D status, calcium-phosphate-PTH profile, bone density or trabecular assessment when indicated, body composition, metabolic-inflammatory context.

### 9.4 Research priorities

Three research priorities follow from this kidney-centered framework. First, longitudinal pediatric cohorts should determine whether endocrine and metabolic measures add predictive information beyong *GLA* genotype, residual α-galactosidase A activity, lyso-Gb3 level, albuminuria, eGFR, blood pressure, symptoms, and family history. Second, female-specific studies should test whether X-linked mosaicism, reproductive history, pregnancy outcomes, thyroid status, bone-mineral markers, and body composition improve prediction of renal trajectories. Third, future registries should distinguish endocrine or metabolic signals that are Fabry-related and actionable from those that represent comorbidity or exploratory associations. This distinction is essential before endocrine and metabolic markers can be incorporated into validated renal risk-stratification models. Recent long-term outcome studies also underscore the need for future surveillance models to integrate renal predictors, genetic classification, biomarker trajectories, treatment exposure, and patient-reported outcomes (Mannan et al., 2025; McCarron et al., 2025).

## 10 Limitations and future validation

Several limitations should be acknowledged. First, the proposed surveillance model has not been prospectively validated. Second, endocrine and metabolic findings in Fabry disease are heterogeneous and may reflect comorbidities, treatment exposure, renal impairment, reduced mobility, or age- and sex-related factors rather than Fabry-specific mechanisms. Third, available studies are often small, cross-sectional, and insufficiently stratified by *GLA* variant, residual α-Gal A activity, lyso-Gb3 level, sex, XCI status, and renal stage. Therefore, this framework should be viewed as a pragmatic tool for risk-adapted monitoring rather than an evidence-based clinical guideline. Prospective cohorts and registries are needed to determine whether these markers improve renal risk stratification or patient outcomes beyond established renal biomarkers.

## 11 Discussion and conclusion

Fabry disease should not be reduced either to a storage disorder or to a generic multisystem disease review. The central question is no longer whether endocrine and metabolic abnormalities occur in Fabry disease. Rather, the key challenge is to determine whether these signals provide actionable information beyond established renal biomarkers and improve longitudinal risk stratification across genetically and clinically heterogeneous patient populations.

The most clinically useful frame is therefore selective rather than encyclopedic. Genetic heterogeneity defines molecular risk; biological modifiers shape penetrance; Fabry nephropathy provides the measurable clinical anchor; and endocrine and metabolic signals are prioritized according to renal relevance and evidence strength.

Endocrine and metabolic assessment should be tested not as a diagnostic substitute, but as a low-burden, risk-adapted longitudinal surveillance layer—and the priority for future cohort and registry studies is to determine which of these signals are actionable, which are comorbid, and which remain exploratory.
